# Novel Bovine Plasma Protein Film Reinforced with Nanofibrillated Cellulose Fiber as Edible Food Packaging Material

**DOI:** 10.3390/membranes12010031

**Published:** 2021-12-27

**Authors:** Shihan Weng, Sara Sáez-Orviz, Ismael Marcet, Manuel Rendueles, Mario Díaz

**Affiliations:** Department of Chemical and Environmental Engineering, University of Oviedo, C/Julian Clavería 8, 33006 Oviedo, Spain; wishwsh@yeah.net (S.W.); saezsara@uniovi.es (S.S.-O.); marcetismael@uniovi.es (I.M.); mariodiaz@uniovi.es (M.D.)

**Keywords:** blood, plasma, protein, nanofibrillated cellulose fiber, packaging

## Abstract

Proteins, such as those in blood from slaughterhouses, are a good option for developing edible films. However, films made exclusively from proteins have low strength and high water solubility, which makes them difficult to use in the food industry. The use of cellulosic material, such as nanofibrillated cellulose (NFC), can improve the properties of these films. In the present work, bovine plasma was acidified and treated with ethanol to precipitate its proteins, and these proteins were used to prepare films reinforced with several concentrations of NFC. In addition, control films prepared with untreated bovine plasma and reinforced with NFC were prepared as well. These new edible films were characterized according to their mechanical properties, water vapor permeability, light transmittance, and microstructure. Furthermore, the film with the best properties was selected to be additivated with nisin to test its antimicrobial properties by wrapping meat previously contaminated with *Staphylococcus aureus*. In this sense, films prepared with the extracted proteins showed better properties than the films prepared with untreated plasma. In addition, the results showed that the reinforcement of the films with a 10% (*w/w*) of NFC decreased their water solubility and improved their puncture strength and water vapor barrier properties. Finally, the addition of nisin to the films prepared with extracted protein from bovine plasma and NFC gave them antimicrobial properties against *S. aureus*.

## 1. Introduction

Nowadays, the use of traditional plastics as packaging material is being replaced by new biodegradable materials, as they are an environmentally friendly alternative [1]. As an advantage, these new materials can be designed to extend the shelf life and improve the quality of foodstuff by controlling different aspects such as water transfer and lipid oxidation [2]. These new edible films and coatings will meet the present and future needs and demands of the food sector.

Of all the possible options for the development of these materials, polysaccharides (as starch or alginate) and proteins (as gelatin and casein) stand out [2,3]. One of the advantages of these materials over synthetic materials is that they can be obtained from co- and by-products from the food industry itself. One of the most problematic co-products of the food industry is blood since it is a major by-product of slaughterhouses and it has a high pollutant capacity [4,5,6]. A total amount of worldwide blood produced from livestock slaughtering of around 4.56 × 10^9^ L [7] has been estimated, considering that 15 L of blood would be obtained from each cattle and 2 to 3 L from each pig [8]. The European Community report calculates similar numbers since they estimate that from each bovine between 10 and 20 L of blood are obtained and from each pig between 2 and 4 L [9]. Therefore, the volumes obtained are remarkably high.

Blood itself has an excellent nutritive value due its high protein content, the rich content of iron, and the bioavailability of its nutrients [5,6,7]. Although there may be some objection to its consumption, it is a good source of nutrients that has been used since ancient times [7]. In addition, in spite of the fact that blood can be processed to generate high-added value food ingredients due to its outstanding functional properties, it is estimated that only 30% of the blood produced in slaughterhouses is employed as food ingredients, mostly as black pudding and similar food products [5].

In this sense, blood can be fractionated into plasma, which represents 65–70% of its content. Blood plasma is rich in proteins (7.9% of protein content [10]), composed mainly of albumin, globulins, and fibrinogen [7]. Some researchers have used plasma proteins as substitutes for other food components. For example, in the production of gluten-free bread (using bovine plasma to improve textural properties [11]), surimi (using plasma proteins as protease inhibitors [12]), or in ham pate (bovine plasma as fat replacer [13,14]). Furthermore, these proteins can used as a matrix for the development of edible films, considerably reducing the environmental and economic impact of this by-product [4,15].

Although films made from proteins generally have good characteristics, they have some limitations, particularly in terms of mechanical strength and hydrophilic characteristics [1,2,3,16]. To address this problem, there are different alternatives to improve their characteristics. Among the most common strategies are chemical and enzymatic modifications and the use of different crosslinking agents [2]. In this sense, nanocellulose and its derivates have recently attracted attention as they can be used as a reinforcement agent in protein films [17,18]. Nanofibrillated cellulose (NFC) is a promising renewable and environmentally friendly material [17,18], which can improve film properties due its characteristics, such as low density and high strength [19]. Thus, the addition of this material to protein films has the potential to develop novel packaging that meets the requirements for a wide range of different food products. In addition, these new types of films can be additivated with different compounds, such as antioxidants or antimicrobials. Among the antimicrobial compounds, nisin stands out. It is a bacteriocin that has antimicrobial activity against a wide range of Gram-positive foodborne pathogens [20]. Nisin affects both cell wall synthesis and the formation of membrane pores [21]. Furthermore, it is the only bacteriocin that is widely employed in the preservation of commercial foodstuff [22].

Therefore, the aim of this research is to develop blood plasma protein-based films with improved properties by adding NFC in their formulation. Firstly, bovine plasma was acidified and treated with ethanol to precipitate its proteins [4], and these proteins were used to prepare films reinforced with several concentrations of NFC. In addition, control films prepared with untreated bovine plasma and reinforced with NFC were prepared. The effect of the protein extraction and the different NFC ratios on the mechanical and physical properties of the films prepared was studied. Finally, the films that showed the best properties were chosen to be additivated with nisin and their antimicrobial properties were tested by wrapping a piece of meat previously contaminated with a common food-borne pathogen such as *Staphylococcus aureus*.

## 2. Materials and Methods

### 2.1. Obtaining Lyophilized Plasma

Blood was collected from a local slaughterhouse (Macelo de Asturias, S.A., Asturias, Spain). As anticoagulant, sodium citrate (2% (*w/v*) (Sigma-Aldrich, Steinheim, Germany)) was used. Plasma was separated from the cell fraction by centrifugation at 10,000× *g* at 10 °C for 10 min. Residual salt components in the plasma were removed using 14 kDa cellulose membranes (Dialysis tubing cellulose membrane, Sigma-Aldrich). Finally, the plasma was frozen at −80 °C for 12 h and then lyophilized (Telstar Cryodos, 0.1 mBar, −70 °C for 24 h).

### 2.2. Acidizing Treatment and Ethanol Extraction of Plasma Protein

In order to obtain acidified plasma protein, the protocol of Álvarez et al. [4] was followed. Briefly, 1.5 g of lyophilized plasma powder were dissolved in 50 mL of distilled water. The pH was adjusted to 2.5 with HCl 3.0 M (Sigma-Aldrich). The acidified plasma was added into 400 mL of 96% ethanol (VWR, Radnor, PA, USA) and pH was adjusted to 1.5 with HCl 3.0 M. The mixture was centrifuged at 10,000× *g* at 10 °C for 30 min and the pellet was stored for further use in film preparation. As stated in the previous work, the main proteins found in the untreated blood plasma were present in the sediment in the same proportion. As control, lyophilized non-acidified plasma was used to develop films as described by Nuthong, Benjakul, and Prodpran [15]. In both cases, the protein content was determined by the Dumas combustion method using a CNHS/O elementar vario EL analyzer (Elementar, Germany).

### 2.3. Preparation of Cellulose Nanofibrillated Fiber (NFC)

Cellulose was bleached by the ECF industrial process from eucalyptus provided by the company ENCE (Navia, Asturias, Spain). Bleached cellulose was subjected to chemical and mechanical treatment to obtain nanofibrillated cellulose fiber (NFC).

To this end, 5 g of compressed cellulose were soaked and softened by adding 400 mL of deionized water and stirring gently for 12 h. In order to oxidize the cellulose, it was treated with TEMPO^®^ (BioMérieux, France). For that purpose, 0.06 g TEMPO^®^ (BioMérieux, France) and 0.6 g NaBr (Sigma-Aldrich) were added to the soaked cellulose pulp. Once they were dissolved, 100 mL of NaClO (6–14% aqueous solution, Merck) was added and the pH was adjusted to 10.0 to start the chemical reaction. The mixture was left 3 h at room temperature and the reaction was stopped by raising the pH to 7.00. Then, the mixture was centrifuged at 10,000× *g* at 8 °C for 1 h and the pellet was washed once with distilled water. Finally, in order to obtain the NFC, the oxidized cellulose was dissolve in 400 mL of distilled water and was homogenized at 15,000 rpm for 15 min employing a Silent Crusher M homogenizer (Hidford, CT, USA). The mixture was centrifugated at 10,000× *g* at 8 °C for 110 min and NFC was obtained in the pellet. The solid content was analyzed using an HR73 Halogen Moisture analyzer (Mettler Toledo, Columbus, OH, USA).

### 2.4. Film Preparation

Films were prepared using protein extracted from acidified plasma treated with ethanol (FA) or lyophilized plasma (FL) in combination with NFC. The film-forming solutions were prepared considering the protein concentration in the lyophilized plasma and in the pellet obtained after treating the bovine plasma, as was described in Section 2.2. The detailed composition of the film-forming solutions is shown in Table 1.

Acidified plasma protein and lyophilized plasma protein were dissolved in distilled water by stirring at 600 rpm for 30 min at room temperature. NFC films (FC) were prepared by adding NFC to the film-forming solution and stirring at 5000 rpm for 5 min. In this case, three different percentages of NFC (10, 30, and 50%) were added to the film-forming solutions prepared with both acidified plasma protein (FA90FC10, FA70FC30, and FA50FC50) and lyophilized plasma (FL90FC10, FL70FC30, and FL50FC50), maintaining the solids’ concentration at 0.030 g/mL for every film-forming solution prepared. In all cases, glycerol (Sigma-Aldrich) was employed as plasticizer and added to the solutions at 70% (*w/w* of solids). All film-forming solutions were cast on silicone molds and were dried at 37 °C for 24 h.

### 2.5. Film Characterization

Prior to testing, all films were placed in a desiccator at room temperature for 24 h. The desiccators maintained a relative humidity of 54 ± 2% and had a saturated solution of Mg(NO_3_)_2_ (Sigma-Aldrich) placed at the bottom.

#### 2.5.1. Thickness and Mechanical Properties

The thickness of the films was measured at five different points, both inside and outside the films. For this purpose, a digital micrometer (Mitutoyo, Kawasaki, Japan) was employed. The film’s thickness reported was the average of these values.

The mechanical properties were tested according to the method described by [14] using TA.XTplus Texture Analyzer (Stable Micro Systems, Godalming, UK) equipped with a 5 kg load cell. Films were cut into squares and placed on the test platform. Samples were subjected to a penetration test at room temperature using a P/5 S probe (5 mm of diameter) and a test speed of 1 mm/s. Puncture strength (PS) and puncture deformation (PD) values were obtained according to the following equations [23]

PS=Fm/Th

$$PD=\left(\sqrt{D^{2}+R^{2}}-R\right)/R$$

where Fm is the maximum force applied before the film was broken (N), Th is the thickness of the film (mm), *D* is the distance covered by the probe in contact with the film until it is broken (mm), and *R* is the radius of the hole in the plates (mm). Experiments were carried out in triplicate and reported results correspond to the mean value.

#### 2.5.2. Light Transmission and Transparency

Visible and ultraviolet (UV) light barrier properties of the films were tested at different wavelengths in the range of 200 to 600 nm [24] using a spectrophotometer (Helios gamma, Thermo Fischer Scientific, Waltham, MA, USA). An empty quartz cuvette was employed as blank, and samples were measured as rectangular pieces of film. The transparency of the film was calculated according to following equation

$$Transparency=A_{600}/X$$

where *A*_600_ is the absorbance of the film sample at 600 nm and *X* is the film thickness (mm). Experiments were carried out in triplicate and reported results correspond to the mean value.

#### 2.5.3. Water Vapor Permeability (WVP)

Undamaged films with no holes were cut into circles with the same diameter as PVC cups filled with distilled water. The films stuck to the glasses, leaving a gap of 1 cm between the water surface and the films. The mounted cups were placed in a room at 20 °C and the weight loss was recorded every hour for the first 10 h and finally after 24 h. The weight loss was plotted against time and the water vapor transmission rate (*WVTR*) was calculated according to the following equation

WVTR=G/(t*A)

where *G/t* is the change in the weight of the cup per unit of time (g/h) and *A* is the area of the cup covered by the film (m^2^).

These *WVTR* values were used to calculate the water vapor permeability (*WVP*) using the following equation

WVP=(WVTR*Th)/ ΔP

where *Th* is the thickness of the film (mm) and ΔP is the water vapor difference across the film (kPa). All experiments were carried out in triplicate and reported results correspond to the mean value.

#### 2.5.4. Water Solubility (WS)

In order to test WS, circular pieces of the films were immersed in 20 mL of distilled water with 2% (*w/w*) of HCl at pH 7.0 (Sigma-Aldrich) and were kept at room temperature for 24 h. After that time, the film pieces were recovered by filtering using a vacuum pump and Whatman N °1 paper, and then dried in an oven at 105 °C for 24 h. Furthermore, pieces of the film were dried at the same conditions without first being dissolved. The solubility was calculated as follows [25]

WS(%)=(m1−m2)/m1×100

where *m*1 is the weight (g) of the film pieces dried in an oven at 105 °C for 24 h, and *m*2 is the weight (g) of the undissolved films pieces once they have been dried. Experiments were performed in triplicate and reported results correspond to the mean value.

#### 2.5.5. Scanning Electron Microscopy (SEM)

The cross-section microstructure of the films was observed by using a scanning electron microscope (SEM) (JSM-6610LV, JEOL, Pleasanton, CA, USA). Lyophilized films were cut into 1 cm^2^ square pieces employing a surgical blade. These samples were mounted on stubs and coated with gold for 5 min in an argon atmosphere. The morphology of the films was observed at magnifications of between 370 and 500× with a voltage of 20 kV.

### 2.6. Antimicrobial Properties of Films Additivated with Nisin

To test the antimicrobial capacity against *Staphylococcus aureus*, nisin was added to FA90FC10 films. *S. aureus* CECT 240 (from the Spanish Type Culture Collection, Valencia, Spain) was grown in 100 mL of TSB (Tryptone Soy Broth, Sigma-Aldrich) in an orbital shaker at 200 rpm and 37 °C for 24 h. Then, 50 g of meat (purchased at a local market) was cut into squares. Each piece was infected with 100 µL of 10^5^ CFU/mL of *S. aureus* in a 0.7% NaCl solution (Sigma-Aldrich). After the liquid had dried, the pieces of meat were coated with films loaded with 3 mg/mL of nisin (1000 IU/mg; Sigma-Aldrich). All pieces were stored in a fridge and the growth evolution of *S. aureus* was analyzed at different times (0, 1, 3, 6, 10, and 15 days). For this purpose, the pieces of meat to be sampled were placed in a Stomacher™ bag (Seward, West Sussex, UK) with 10 mL of NaCl 0.7% (*w/v*) and were homogenized with a Stomacher™ device (Seward, UK) at maximum speed for 120 s. Microbial growth was analyzed by preparing serial dilutions (1:10) and incubating on Baird-Parker medium enriched with egg yolk tellurite emulsion (both from Sigma-Aldrich) with 2% of agar (VWR) plates for 48 h at 37 °C. Each sample was carried out in triplicate and results were expressed in log_10_ CFU/g of meat.

### 2.7. Statistical Analysis

All experiments were performed in triplicate and results are shown as the mean value. To analyse differences between the groups tested, analysis of variance (ANOVA) was carried out. Fischer’s Least Significant Difference (LSD) was used to determine significant differences between the groups. A level of *p* < 0.05 was considered significant. Analyses were performed using IBM^®^ SPSS^®^ Statistics V25 statistical software.

## 3. Results and Discussion

### 3.1. Film Characterization

#### 3.1.1. Thickness and Mechanical Properties

The thickness and mechanical properties of the films were analysed, and the results are shown in Table 2. Regarding thickness, the films ranged from 123 to 176 µm. Significant differences (*p* < 0.05) were detected between the films according to their composition, with the mixture of FLFC films being the thinnest.

Concerning the mechanical properties, significant differences were found between the FA and FL films (Table 2). The PS values were notably different, with a mean value of 68.3 N/mm for FA100 films and 23.4 N/mm for FL100 films. Therefore, the treatment to which the bovine plasma protein was subjected affected the mechanical properties of the films. This treatment causes an exposure of the hydrophobic cores of the plasma proteins by their partial denaturalization [4]. In this way, treated proteins have a high capacity for forming hydrophobic bonds, increasing the PS values of these films

Regarding the addition of NFC, the best PS result was obtained for the FA90FC10 films, where the value of this parameter noticeably increased. With the addition of a higher amount of NFC, the PS values started to decrease in all cases. The use of NFC as a reinforcement in protein films is being widely studied and similar results were obtained when “faba” bean protein [3] and fish myofibrillar protein [26] were employed. Hydroxyl groups of NFCs have the capacity to interact with the hydrophilic groups of the proteins in the film matrix by forming hydrogen bonds [26,27], which increases the cohesion of the biopolymers and improves the mechanical properties of the materials prepared [3,17]. However, an excessive addition of NFC could lead to its uneven distribution, resulting in an inhomogeneous film due to the agglomeration of the modified cellulose, and, hence, in the production of films with poor mechanical properties [17].

In the case of the elasticity of the films, the addition of NFC decreased the values of the PD parameter (Table 2). This decrease in the PD values is usual in protein-based films reinforced with cellulose fibers, and the same behavior has been reported by several authors [27,28]. The presence of cellulose derivatives reduces the mobility of the polymer due to an increase in interactions with the proteins in the matrix, leading to a loss of elasticity and ductility of the films [26].

#### 3.1.2. Light Transmittance and Transparency

**Figure 1:**
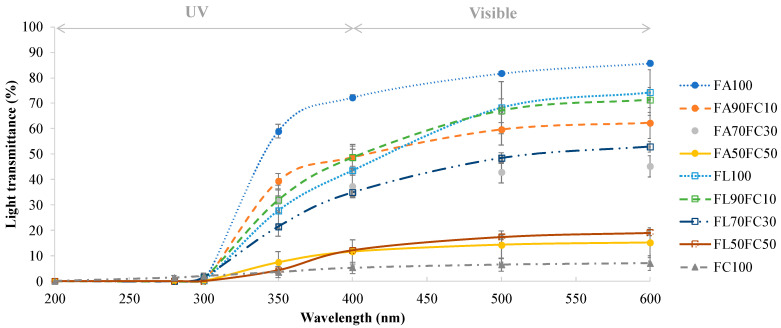
Light transmittance (%) of the films at different wavelengths (200–600 nm). FA refers to acidified plasma protein, FL to lyophilized plasma protein and FC to NFC. The number refers to the percentage of each component added to prepare the film. ANOVA and LSD were performed. Significant differences (*p* < 0.05) were found between four different groups (FA100, FL100-FA90FC10-FL90FL10, FA70FC30-FL70FC30, and FC100-FA50FC50-FL50FC50).

The light transmittance of the films ranged between 200 and 600 nm (Figure 1). For UVC and UVB regions (200–280 nm and 280–315 nm, respectively), the transmittance of all of the samples was very low. In the UVA range (315–400 nm), there was an increase in the optical transmittance in the samples that continued in the visible region (400–600 nm). Low transmittance in the UV range is a desirable property, as this radiation is one of those responsible for the formation of free radicals in lipid-rich foodstuff and its degradation by oxidation processes [29,30]. Thus, films developed using proteins contain aromatic amino acids in their composition, such as tyrosine and tryptophan, that are capable of absorbing the UV light [31]. Significant differences (*p* < 0.05) were found between the samples in the visible region. As the proportion of NFC added increased, the values of transmittance were lower. Therefore, the lowest transmittance values in the visible region were obtained for FC100 films (7.16%) and FA50FC50-FL50FC50 films (15.23% and 19.07%, respectively). Other authors obtained similar results in terms of light transmittance by adding NFC to protein films [3,26,32]. This low transmittance could be attributed to the presence of Xylan since it is supposed to interfere partially with the complete dispersion of the nanofibrils in water [19] and to the distribution of the nanofibers in the polymer substrate as they form their own network [26]. For films developed with protein extracted from acidified plasma and untreated lyophilized plasma, no significant differences were observed (*p* > 0.05). Therefore, this treatment did not affect the values of light transmittance.

Regarding the transparency index of the films, results are shown in Table 1. According to the results obtained when the light transmittance was measured in the visible region, the most transparent films were those made exclusively from plasma protein (FA100 and FL100). As the concentration of NFC added to the films increased, the transparency index values also increased, so the films became opaquer. This opacity is caused by the light scattering by the cellulose nanofibers, causing a reduction in the light passing through [26], and this has also been observed by other authors [3,19,33]. In this case, the reinforcement of 10% NFC was the most adequate, as a high degree of transparency was not lost.

#### 3.1.3. Water Vapor Permeability (WVP) and Water Solubility (WS)

WVP results are reported in Table 3. Significant differences were found between FA and FL samples, so the treatment improved the water vapor barrier properties. In addition, the increase in hydrophobic and hydrophilic interactions between the NFC and the proteins promoted a decrease in the permeability of the water vapor across the films tested [26]. Furthermore, the low permeability of these films added with NFC can be enhanced by the high degree of crystallization of cellulose and its ability to form a dense percolating network [19]. The results obtained are similar to those of other authors who have reinforced films with CNF in alginate [17] and sodium caseinate matrices [28]. However, other researchers have found that an excess of cellulosic material can lead to increased WPV values due to an accumulation of NFC and the loss of a well compacted structure [17].

Regarding the WS of the films, results are shown in Table 3. Films made exclusively with lyophilized bovine plasma showed a high degree of solubility (FL100), but films made with extracted proteins from acidified plasma were highly insoluble (FA100). Acid treatment and ethanol precipitation could be responsible for the variation in solubility [4]. This would be a key factor for their use in the food field as most foodstuffs have a high moisture content and the films would be water resistant [2]. The addition of NFC allowed improvements to be made to the characteristics of the films as the solubility values decreased as the percentage of NFC added was increased. The strong interactions of hydrophobic and hydrogen bonds between the NFC surface and proteins make the films more consistent and, therefore, reduce the sensitivity to water molecules [26,34]. Other authors have explained this lower water solubility due to the high level of crystallization of NFC [35]. As for films with other CNF-reinforced protein matrices [3,26], acidified plasma protein films showed a similar WS. Furthermore, the addition of NFC made it possible to reduce further the solubility of the films produced, which improves their performance and range of applications as they can be used in a greater number of foodstuffs.

#### 3.1.4. Visual Appearance and Scanning Electron Microscopy (SEM)

**Figure 2:**
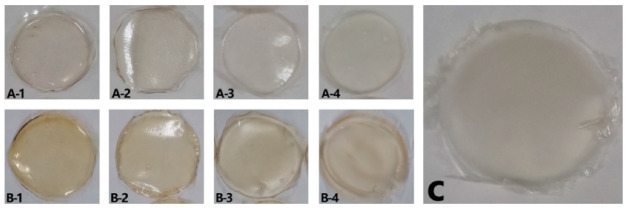
Visual appearance of the films. (**A-1**) FA100, (**A-2**) FA90FC10, (**A-3**) FA70FC30, (**A-4**) FA50FC50, (**B-1**) FL100, (**B-2**) FL90FC10, (**B-3**) FL70FC30, (**B-4**) FL50FC50, and (**C**) FC100. FA refers to acidified plasma protein, FL to lyophilized plasma protein, and FC to NFC. The number refers to the percentage of each component added to prepare the film.

The visual appearance of the films is shown in (Figure 2). They were all homogeneous although films made directly from lyophilized bovine plasma (FL) showed a more yellowish color than those made with protein extracted from acidified plasma protein (FA). This is due to the presence of yellowish compounds in the bovine plasma, such as bilirubin [36], carotenoids [37], and hemoglobin [38]. These compounds were removed by the acid-ethanol treatment [4] and, hence, the FA films obtained were colorless.

In addition, as the proportion of NFC in the film composition increased, the films became opaquer, as was observed when the transparency index values were calculated (Table 1). Therefore, the most transparent films were those that did not contain NFC in their composition (FA100 and FL100). In addition, transparency may be considered good even for films containing 30% NFC, as they were not so opaque that foodstuff could not be inspected.

**Figure 3:**
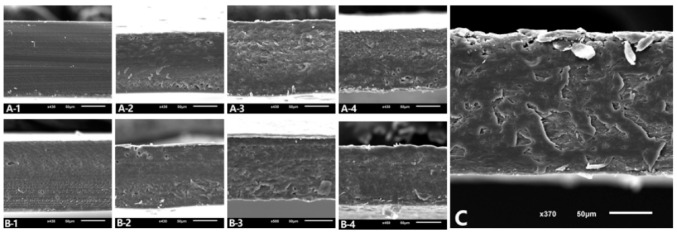
SEM images of the cross-section of the films. Scale bars correspond to 50 µm. (**A-1**) FA100, (**A-2**) FA90FC10, (**A-3**) FA70FC30, (**A-4**) FA50FC50, (**B-1**) FL100, (**B-2**) FL90FC10, (**B-3**) FL70FC30, (**B-4**) FL50FC50, and (**C**) FC100. FA refers to acidified plasma protein, FL to lyophilized plasma protein, and FC to NFC. The number refers to the percentage of each component added to prepare the film.

Regarding microstructure, micrographs of the cross-section are shown in Figure 3. FA100 and FL100 films showed a homogeneous and uniform appearance. The addition of a high percentage of NFC (30% or above) led to some changes in the microstructure, becoming less homogeneous, with agglomerations inside the matrix. This may be due to the natural tendency of cellulosic fillers to self-associate via hydrogen bonds as their concentration increases [39]. In addition, it should be noted that there was a decrease in the amount of protein that could interact with the film itself, which make these materials less compact and weaker. Moreover, at an NFC concentration of 10%, no changes in microstructure were observed, suggesting the optimal interfacial adhesion between the protein matrix and the cellulose reinforcement. This result agreed with the mechanical properties of the films (Section 3.1) as FA90FC10 and FL90FC10 showed the best strength results in their group.

### 3.2. Antimicrobial Properties of Films Additivated with Nisin

**Figure 4:**
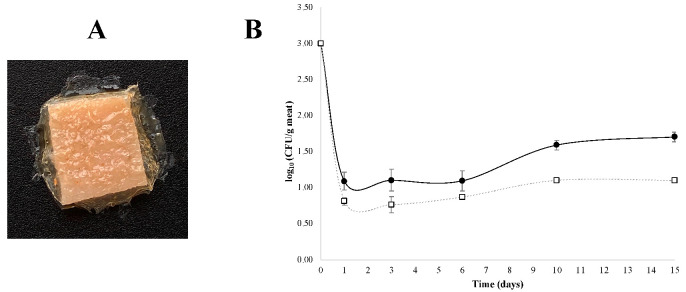
(**A**) Visual appearance of pieces of meat wrapped with FA90FC10 films additivated with nisin. (**B**) Evolution of *S. aureus* growth in meat for 15 days storage at 4 °C; (□) FA90FC10 films additivated with nisin and (●) FA90FC10 control films without nisin. ANOVA and LSD were performed. Significant differences between samples were found from day 6.

For the antimicrobial tests, FA90FC10 films were selected as they showed the best properties in the previous analyses. To test the inhibitory effect on a real food model, pieces of meat were wrapped with these dried films additivated with nisin. As can be shown in Figure 4A, there were no breaks, and the films were in contact with the whole surface of the previously contaminated piece of meat. The effect in the growth of *S. aureus* was analysed over time and results are shown in Figure 4B.

In both cases, there was a rapid decrease in *S. aureus* concentration (of at least two logarithmic units) during the first day of storage (Figure 4B). This could be explained by the sudden change in environmental conditions and temperature as the bacteria were transferred from 30 °C in a laminar flow hood to 4 °C in a fridge. From day one onwards, the concentration of *S. aureus* began to grow, and significant differences were observed between the control and nisin-additivated films from day 6. After 15 days, the final concentration of *S. aureus* was 1.70 log_10_ CFU/g of meat for the control films and 1.00 log_10_ CFU/g of meat for the nisin-additivated FA90FC10 films. In both cases, the concentrations were below the recommended limit in meat products (10^3^ CFU/g or mL of food product) [40,41].

Therefore, the films developed in this research showed good characteristics and can be additivated with different antimicrobial compounds. This would make it possible to extend the shelf life of perishable food products or foodstuff susceptible to contamination during the production and transport process. For the specific case of *S. aureus*, it is commonly detected in raw meat, but the main problem is that it can be transmitted most often via food handlers and in the food production chain [42,43]. Thus, the development of films that are able to control its growth and proliferation in foodstuffs is a key factor.

## 4. Conclusions

Films prepared with ethanol-extracted proteins after acidifying bovine plasma and being reinforced with NFC showed better characteristics than those observed in the control films prepared with untreated plasma. However, when NFC was incorporated into these films in high concentrations, it worsened certain characteristics, such as their mechanical properties. Therefore, NFC has limited capacity as a reinforcement in this type of film. After the characterization of all of the films developed, it was found that the optimum formulation was FA90FC10. These films, formulated with 10% NFC, showed a significant improvement in their mechanical and water vapor barrier properties, whilst their water solubility decreased, without compromising their optical properties such as the light transmittance and the transparency index. In addition, it was found that these films can be easily formulated with antimicrobial compounds such as nisin. A more detailed study of the packaging possibilities of these materials is needed to check other aspects, such as which type of application is the most suitable in foodstuffs (coatings or films) or their possible antioxidant capacity.

## Figures and Tables

**Table 1:** Composition of the film-forming solutions prepared using acidified plasma protein, lyophilized plasma, and nanofibrillated cellulose (NFC). FA refers to films prepared with acidified plasma protein, FL to films prepared with lyophilized plasma, and FC to films prepared with NFC.

Film	Acidified Plasma Protein (g/mL)	Lyophilized Plasma Protein (g/mL)	NFC (g/mL)
FA100	0.030	-	-
FA90FC10	0.027	-	0.003
FA70FC30	0.021	-	0.009
FA50FC50	0.015	-	0.015
FL100	-	0.030	-
FL90FC10	-	0.027	0.003
FL70FC30	-	0.021	0.009
FL50FC50	-	0.015	0.015
FC100	-	-	0.030

**Table 2:** Puncture strength (PS), puncture deformation (PD), and thickness of the films. ANOVA and LSD were performed. Different letters in the same column indicate significant differences (*p* < 0.05).

Film	PS (N/mm)	PD (%)	Thickness (µm)	Transparency
FA100	68.3 ± 4.4 ^a^	34.8 ± 5.0 ^a^	167 ± 12 ^a^	0.45 ± 0.06 ^de^
FA90FC10	75.6 ± 3.0 ^b^	25.4 ± 6.1 ^b^	172 ± 20 ^a^	1.25 ± 0.17 ^cd^
FA70FC30	55.4 ± 0.7 ^c^	13.1 ± 1.6 ^cd^	161 ± 24 ^ab^	2.11 ± 0.24 ^b^
FA50FC50	21.0 ± 2.2 ^d^	10.1± 1.5 ^d^	158 ± 19 ^ab^	5.32 ± 0.20 ^a^
FL100	23.4 ± 2.1 ^d^	15.7 ± 5.3 ^bc^	176 ± 47 ^a^	0.64 ± 0.10 ^de^
FL90FC10	46.3 ± 6.2 ^c^	17.6 ± 6.0 ^bcd^	140 ± 28 ^bc^	1.19 ± 0.28 ^cd^
FL70FC30	32.5 ± 2.7 ^e^	17.7 ± 6.6 ^bc^	144 ± 10 ^bc^	1.91 ± 0.10 ^bc^
FL50FC50	29.6 ± 6.3 ^e^	12.4 ± 1.2 ^cd^	123 ± 4 ^c^	5.65 ± 0.17 ^a^
FC100	30.5 ± 1.1 ^e^	8.8 ± 3.7 ^d^	159 ± 20 ^a^	5.84 ± 0.14 ^a^

FA refers to films prepared with acidified plasma protein, FL to films prepared with lyophilized plasma, and FC to films prepared with nanofibrillated cellulose. The number refers to the percentage of each component added to prepare the film.

**Table 3:** Water vapor permeability (WVP) and water solubility (WS) of the films. ANOVA and LSD were performed. Different letters in the same column indicate significant differences (*p* < 0.05).

Film	WVP (g × mm/m^2^h × kPa)	WS (%)
FA100	3.81 ± 0.10 ^a^	14.40 ± 2.31 ^a^
FA90FC10	2.92 ± 0.11 ^b^	11.32 ± 0.72 ^b^
FA70FC30	2.87 ± 0.10 ^b^	10.50 ± 1.60 ^b^
FA50FC50	2.72 ± 0.12 ^b^	7.10 ± 1.00 ^c^
FL100	4.10 ± 0.16 ^c^	99.60 ± 0.20 ^d^
FL90FC10	3.80 ± 0.53 ^a^	88.70 ± 3.23 ^e^
FL70FC30	3.23 ± 0.15 ^d^	75.25 ± 0.41 ^f^
FL50FC50	2.58 ± 0.17 ^e^	64.03 ± 2.36 ^g^
FC100	2.75 ± 0.18 ^e^	14.40 ± 2.31 ^a^

FA refers to films prepared with acidified plasma protein, FL to films prepared with lyophilized plasma, and FC to films prepared with nanofibrillated cellulose. The number refers to the percentage of each component added to prepare the film.

## Data Availability

Data are contained within the article.
